# The impact of growth at elevated [CO_2_] on stomatal anatomy and behavior differs between wheat species and cultivars

**DOI:** 10.1093/jxb/erad011

**Published:** 2023-01-12

**Authors:** Shellie Wall, James Cockram, Silvere Vialet-Chabrand, Jeroen Van Rie, Alexander Gallé, Tracy Lawson

**Affiliations:** School of Life Sciences, University of Essex, Colchester CO4 3SQ, UK; NIAB, 93 Lawrence Weaver Road, Cambridge CB3 0LE, UK; School of Life Sciences, University of Essex, Colchester CO4 3SQ, UK; BASF Belgium Coordination Center CommV-Innovation Center Gent, Technologiepark-Zwijnaarde 101, 9052 Gent, Belgium; BASF Belgium Coordination Center CommV-Innovation Center Gent, Technologiepark-Zwijnaarde 101, 9052 Gent, Belgium; School of Life Sciences, University of Essex, Colchester CO4 3SQ, UK; MPI of Molecular Plant Physiology, Germany

**Keywords:** Bread wheat, net CO_2_ assimilation rate (*A*), stomatal density, stomatal conductance (*g*_s_), *Triticum aestivum* L, wheat relatives (*Aegilops tauschii*, *Triticum turgidum* ssp*. dicoccoides*, *Triticum turgidum* ssp. *dicoccon*)

## Abstract

The ability of plants to respond to changes in the environment is crucial to their survival and reproductive success. The impact of increasing the atmospheric CO_2_ concentration (a[CO_2_]), mediated by behavioral and developmental responses of stomata, on crop performance remains a concern under all climate change scenarios, with potential impacts on future food security. To identify possible beneficial traits that could be exploited for future breeding, phenotypic variation in morphological traits including stomatal size and density, as well as physiological responses and, critically, the effect of growth [CO_2_] on these traits, was assessed in six wheat relative accessions (including *Aegilops tauschii*, *Triticum turgidum* ssp. *Dicoccoides*, and *T. turgidum* ssp. *dicoccon*) and five elite bread wheat *T. aestivum* cultivars. Exploiting a range of different species and ploidy, we identified key differences in photosynthetic capacity between elite hexaploid wheat and wheat relatives. We also report differences in the speed of stomatal responses which were found to be faster in wheat relatives than in elite cultivars, a trait that could be useful for enhanced photosynthetic carbon gain and water use efficiency. Furthermore, these traits do not all appear to be influenced by elevated [CO_2_], and determining the underlying genetics will be critical for future breeding programmes.

## Teaser

Elevated [CO_2_] affects stomatal characteristics differently in wheat elite cultivars and wild accessions, resulting in faster kinetics in wild relatives, a trait useful to enhanced photosynthesis and water use.

## Introduction

Prior to the industrial revolution, the atmospheric CO_2_ concentration (a[CO_2_]) was maintained at a value close to 280 ppm for ~1000 preceding years (Tans and Keeling, 2016). Subsequently, anthropogenic CO_2_ emissions, primarily through the burning of fossil fuels, have increased the present day atmospheric CO_2_ concentration to 419 ppm (NOAA, 2022). With the current increases in CO_2_ emissions associated with modern day activities, the Intergovernmental Panel on Climate Change projections include scenarios of [CO_2_] doubling from current levels by the end of the century (IPCC, 2021), which to date has resulted in a rise in global temperature (of ~1.1 °C, World Meteorological Organization, 2022) and is predicted to rise with further increases in [CO_2_] (Stockwell *et al.*, 2021). Elevated [CO_2_] generally increases leaf photosynthetic rates in a range of C_3_ crops from potatoes (Lawson *et al.*, 2001) to soybean (Rogers *et al.*, 2004), through increased substrate for Rubisco (the enzyme involved in the first major step of carbon fixation) and the suppression of photorespiration. A recent review of 18 C_3_ crops grown using free-air CO_2_ enrichment (FACE) technology with elevated CO_2_ of 200 ppm above ambient [CO_2_] reported that most species exhibited increased yields by ~18% (Ainsworth and Long, 2021). However, the same study also highlighted that yield increases were not consistent across species or cultivars, and yield benefits with elevated [CO_2_] were correlated with sink strength (Ainsworth and Long, 2021).

As stomatal conductance (*g*_s_) regulates gas exchange between the leaf interior and the external environment, stomatal responses to changing climatic conditions are critical in determining CO_2_ supply for photosynthesis (*A*) and water loss through transpiration (Lawson *et al.*, 1998; Morison *et al.*, 2008). Transpirational water loss also plays a key role in nutrient uptake from the plant roots as well as evaporative cooling of the leaf tissue and the maintenance of optimal leaf temperatures for photosynthesis (Raven, 1977, 2002; Hetherington and Woodward, 2003; Peterson *et al.*, 2010; McAusland *et al.*, 2016; Murray *et al.*, 2016; Lawson and Vialet-Chabrand, 2019). Therefore, stomatal dynamics will have a pivotal role in determining C_3_ crop productivity in future climates (Lawson *et al.*, 2010, 2012).

*g*_s_ is determined by anatomical features as well as functional aspects of the guard cells, both of which are influenced by growth [CO_2_] (and temperature) (Woodward, 1987; Matthews and Lawson, 2019; Stevens *et al.*, 2021). Both stomatal anatomy and behavior are modified by elevated [CO_2_], with most species responding by decreasing stomatal density (Woodward, 1987; Poole *et al.*, 1996) and reducing aperture (see review by Stevens *et al.*, 2021). Reducing stomatal aperture under elevated [CO_2_] greatly increases intrinsic water use efficiency (WUEi; *A*/*g*_s_) with potential benefits for plant growth (Leakey *et al.*, 2009; Sreeharsha *et al.*, 2015). On the other hand, reductions in *g*_s_ can negatively impact on photosynthesis through diffusional constraints, as well increases in leaf temperature (Matthews and Lawson, 2019).

The number and size of stomata on the leaf determine the maximum potential stomatal conductance (*g*_smax_; Lawson and Morison 2004; Lawson *et al.*, 2010; McElwain *et al.*, 2016), whilst pore aperture/behavior regulates the short-time scale dynamics of *g*_s_ and gas exchange (Dow *et al.*, 2014; Takahashi *et al.*, 2015). The majority of studies that have explored variation in *g*_s_ or the influence of growth conditions on anatomy and function have examined steady-state conditions (Schlüter *et al.*, 2003; Doheny-Adams *et al.*, 2012; Tanaka *et al.*, 2013); however, recent studies have illustrated the significant impact of dynamic *g*_s_ responses on *A* (Sakoda *et al.*, 2020, 2022; Vialet-Chabrand and Lawson, 2020) and WUEi (Papanatsiou *et al.*, 2019; Acevedo-Siaca *et al.*, 2021; Pignon *et al.*, 2021). Generally, slow stomatal opening limits the CO_2_ assimilation rate, reducing the speed of photosynthetic induction (Long *et al.*, 2022), whilst slow closure erodes WUEi (Lawson and Blatt, 2014; McAusland *et al.*, 2016; Qu *et al.*, 2016, 2020; Lawson and Vialet-Chabrand, 2019). The rapidity of stomatal responses to changing climatic conditions is also critically important for maintaining optimal leaf temperature (Matthews and Lawson, 2019; Stevens *et al.* 2021) and for photosynthesis and plant productivity (Moore *et al.*, 2021). Dynamic responses have been linked to both morphological and physiological variation in stomata (Drake *et al.*, 2013; Lawson and Blatt, 2014; Zhang *et al.*, 2019); however, few studies have explored the impact of changing climate conditions such as growth [CO_2_] on morphophysiological characteristics. Consequently, natural variation in rapidity of stomatal conductance between cultivars and species, as well as the influence of changing climatic conditions on these traits, could provide currently unexploited targets for improving crop productivity in future climates (Lawson *et al.*, 2012; Faralli *et al.*, 2019; Faralli and Lawson, 2020).

Here we explored the impact of elevated CO_2_ concentration (e[CO_2_]) of 800 ppm, approximately double that of the current atmospheric [CO_2_] (a[CO_2_]), on the physiology and growth of 11 different wheat progenitor and elite cultivar accessions. Wheat (*Triticum aestivum* L.) is a principal global food grain source, grown on more land area than any other commercial crop. In addition, it is one of the largest traded primary crop commodities, along with maize and rice (FAO, 2014). Globally, wheat provides >20% of the calories consumed by the human population (Braun and Atlin, 2010; Lobell *et al.*, 2011). Modern wheat is a hexaploid species containing three sets of chromosomes (A, B, and D subgenomes). These subgenomes originated from three different diploid grass species and combined during two hybridization events (Kerber and Rowland, 1974; Faris, 2014; Marcussen *et al.*, 2014). Initially diploid wheat *Triticum urartu* (subgenome AA ancestor) hybridized with the B genome ancestor *Aegilops speltoides* ssp*. ligustica* (Huang *et al.*, 2002; Dvorak and Akhunov, 2005; Peng *et al.*, 2011) to produce wild emmer wheat *Triticum turgidum* ssp. *dicoccoides* (genome AABB). In the second event, *T. turgidum* ssp. *dicoccoides* hybridized with the wild goat grass *Aegilops tauschii* to produce the modern hexaploid *Triticum aestivum* ssp. *aestivum* (AABBDD; Huang *et al.*, 2002; Charmet, 2011; Faris, 2014). In this study, phenotypic variation in morphological traits including stomatal size and density, as well as physiological responses and, critically, the effect of growth [CO_2_] on these traits, was assessed in six wheat relative (WR) accessions (including the species *Aegilops tauschii*, *T. turgidum* ssp. *dicoccoides*, and *T. turgidum* ssp*. dicoccon*) and five elite wheat *T. aestivum* cultivars (Claire, Rialto, Robigus, Soissons, and Xi19) to identify possible beneficial traits that could be exploited for future breeding.

## Materials and methods

### Plant growth conditions

*Triticum* and *Aegilops* species (listed in Table 1) were germinated in a greenhouse compartment (at BASF, Ghent, Belgium) with supplementary lighting (Master Greenpower CGT 400 W E40 HPS lights) to ensure a typical summer day length of 15.30 h. At 14 d post-emergence, plants were vernalized in a controlled environment (custom-made growth chamber, BASF, Ghent, Belgium) for 10 weeks at 4 °C, with 75 μmol m^–2^ s^–1^ PPFD, over a 10 h photoperiod using an in-house-produced 60/40 peat-based sowing and cutting soil (including NPK Compound Fertilizer 12-14-24 (0.8 kg m^–3^). Plants were then transferred into 4 liter pots using a peat-based, boron-free potting soil [including NPK Compound Fertilizer 12-14-24 (2 kg m^–3^)] and grown in two separate growth environments, one at current (2018) atmospheric [CO_2_] (408 ppm CO_2_) and a second at an elevated [CO_2_] of 800 ppm. Both growth chambers had a light intensity (at pot height) of 800 ± 20 µmol m^–2^ s^–1^ with a 2:1 high pressure sodium:metal halide lighting mix (Master Greenpower CGT 400 W E40 and Powerstar HQI-BT 400 W/D PRO 400 W Daylight E40, respectively) for a 15 h light/9 h dark photoperiod. With the exception of [CO_2_], both growth environments were set to identical conditions: air temperature controlled to 20 °C and 18 °C (±1 °C) day and night, respectively, and relative humidity maintained at a constant 65%. Plants were well watered using a drip irrigation system to the roots. All wheat measurements were taken from the flag leaf, at Zadoks growth stage 49 (GS 49, first awns/scurs visible) to GS 59 (ear emergence complete) (Zadoks *et al.*, 1974). Six repetitions of each measurement were completed per accession unless stated below.

**Table 1.** Species investigated, including ploidy and common name

Species/cultivar abbreviation	Species	Common name	Ploidy
Claire	*Triticum aestivum*	Common or bread wheat	Hexaploid
Rialto	*Triticum aestivum*	Common or bread wheat	Hexaploid
Robigus	*Triticum aestivum*	Common or bread wheat	Hexaploid
Soissons	*Triticum aestivum*	Common or bread wheat	Hexaploid
Xi19	*Triticum aestivum*	Common or bread wheat	Hexaploid
TRI 11502	*Triticum dicoccoides*	Wild emmer	Tetraploid
TRI 3432	*Triticum dicoccon*	Emmer	Tetraploid
IG 48509	*Aegilops tauchii*	Goat grass or rough-spike hard grass	Diploid
IG48514	*Aegilops tauchii*	Goat grass or rough-spike hard grass	Diploid
KU2018	*Aegilops tauchii*	Goat grass or rough-spike hard grass	Diploid
KU 2036	*Aegilops tauchii*	Goat grass or rough-spike hard grass	Diploid

Species abbreviation is how the species is referred to in the text. All seeds were provided from the NIAB collection.

### Leaf anatomical measurements

#### Measurements of stomatal density and size

Stomatal density (SD) was measured from impressions taken from both the adaxial (upper) and abaxial (lower) leaf surface using silicone impression material (Xantopren, Heraeus, Germany) following the methods of Weyers and Johansen (1985) using six leaves per species/cultivar, measured at the middle of the leaf lamina. SD, guard cell length (GCL; used as a proxy for stomatal size), and pore length (PL) were all measured via light microscopy (Olympus BX60, Essex, UK). Total magnification was 100-fold for SD measurements and 400-fold for GCL and PL measurements.

Anatomical maximum stomatal conductance (*g*_smax_: mol m^–2^ s^–1^) was calculated from the measurements of SD and stomatal dimensions (Equation 1) following the equations of Franks and Farquhar (2001):


$$\begin{array}{l} \left(d\;\times \;SD\times a_{max}\right)/\{v\times [l+(\pi /2)\times \surd (a_{max}/\pi )]\} \end{array}$$ (1)

Where *d* is the diffusivity of water in air (m^2^ s^–1^, at 22 °C), *v* is the molar volume of air (m^3^ mol^–1^, at 22 °C), and pore depth (*l*; μm) was equal to guard cell width at the centre of the stoma represented as half the GCL. The mean maximum stomatal pore area (*a*_max_; μm^2^) was calculated assuming stomatal pores were elliptical with the major axis equal to pore length and the minor axis equal to half pore length (see McElwain *et al.*, 2015).

#### Leaf thickness

Leaf thickness (LT) measurements were taken using the MultispeQ v1.0 instrument (Michigan State University, MI, USA) (Kuhlgert *et al.*, 2016). The device was calibrated using 0.18 mm thick filter paper (Whatman 1001-110, Maidstone, Kent, UK). A mean leaf thickness was calculated from three repeat measurements per leaf from three separate leaves per species/cultivar.

#### Dry weight and leaf area

Leaf area was measured using a bench-top area meter (LI-3100C, Li-Cor, Lincoln, NE, USA) where the mean leaf area was calculated from three repeat measurements per leaf from three separate leaves per species/cultivar. Leaves were then placed in paper bags and dried at 60 °C to constant weight and measured using a four-digit balance (Kern, Northamptonshire, UK).

### Leaf gas exchange

Stomatal conductance to water vapor (*g*_s_) and the rate of photosynthetic CO_2_ assimilation (*A*) were measured using a portable gas exchange system (Li-Cor 6400XT, Li-Cor) with an integrated light source (Li6400-40, Li-Cor), consisting of blue and red light-emitting diodes. Leaf temperature and VPD were controlled to 22 °C and 1 ± 0.2 kPa, respectively, throughout the measurements. Gas exchange measurements had a constant flow rate set at 300 μmol s^–1^, with cuvette conditions maintained at a CO_2_ concentration of 400 μmol mol^–1^ (for both plant growth CO_2_ treatments). Gas exchange analysis was completed within the first 7 h of the photoperiod, to minimize any diurnal effects on stomatal opening and photosynthetic activation. All measurements were conducted on the mid-point of fully expanded flag leaves, before anthesis (GS 49–59) (Zadoks *et al.*, 1974). Intrinsic water use efficiency was calculated as WUEi=*A*/*g*_s_. Between five and seven repetitions of each measurement were completed per accession for gas exchange data.

### PPFD step measurements

To measure the response of *A* and *g*_s_ to a single step increase in PPFD, leaves were equilibrated at a PPFD of 100 μmol m^–2^ s^–1^ until both *A* and *g*_s_ were at steady state (defined as <2% change in rate over 5 min). Measurements were made at 30 s intervals, for 10 min at 100 μmol m^–2^ s^–1^, after which PPFD was increased in a single step to 1000 μmol m^–2^ s^–1^ and recorded for a further 60 min. Leaf temperature (Ti), VPD, and [CO_2_] were all maintained at 22 °C, 1 ± 0.2 kPa, and 400 µmol mol^–1^, respectively, throughout the measurement. These data were used to model the response of *A*, *g*_s_, and WUEi to changes in PPFD.

### Intracellular CO_ 2_ response curves (*A*/*C*_ i_)

*A*/*C*_i_ response curves [net CO_2_ assimilation rate (*A*) to intercellular CO_2_ concentration (*C*_i_)] were measured at 1500 μmol m^–2^ s^–1^ PPFD. Photosynthesis was initially stabilized for a minimum of 15 min at 400 μmol mol^–1^, then decreased and measured at 250, 150, 100, and 50 μmol mol^–1^ before returning to the initial value of 400 μmol mol^–1^, and increased to 550, 700, 900, 1100, 1300, and 1500 μmol mol^–1^. Photosynthesis was measured at each [CO_2_] after ~3 min. Leaf temperature and VPD were controlled to 22 °C and 1 ± 0.5 kPa, respectively.

### Modeling gas exchange parameters

The maximum velocity of Rubisco for carboxylation (*V*_cmax_) and the maximum rate of electron transport demand for ribulose bisphosphate dehydrogenase (RuBP) regeneration (*J*_max_) were calculated from the *A*/*C*_**i**_ response using equations from von Caemmerer and Farquhar (1981), as described by Sharkey *et al.* (2007) using the Rubisco kinetic constants for wheat (Carmo-Silva *et al.*, 2010). The response of *g*_s_ to the step change in PPFD was analyzed following the method described in McAusland *et al.* (2016). In summary, the optimum function in R (www.r-project.org; version 3.5.3), a model representing *g*_s_ as a function of time, was fitted on each observed response as shown in Equation 2:


$$\begin{array}{l} g_{s}=\left(g_{smax}-r_{0}\right)e^{-e^{\left(\frac{\lambda -t}{k}+1\right)}}+r_{0} \end{array}$$ (2)

The model uses a sigmoidal equation rather than an exponential slope, with an initial time lag (the time before *g*_s_ starts to increase, λ, min), a time constant (the time taken to reach 63% of the variation, *k*, min), an initial value (*r*_0_, mol m^−2^ s^−1^), and a steady-state target [the value when the plateau is reached (*g*_smax_, mol m^−2^ s^−1^]. The time was set to 0 when PPFD was increased from 100 μmol m^−2^ s^−1^ to 1000 μmol m^−2^ s^−1^ (Vialet-Chabrand *et al.*, 2013).

### Statistical analysis

All statistical analyses were conducted using R software (www.r-project.org; version 3.5.3). For SD, GCL, and *g*_smax_, a Shapiro–Wilk test was used to test for normality and a Levene’s test of homogeneity was used to determine if samples had equal variance. A log transformation was applied when data were not normally distributed (*P*<0.05, Shapiro–Wilk test) to achieve normality and meet modeling assumptions of an ANOVA. Single factor differences were analyzed using *t*-tests with a Bonferroni–Hochberg end correction or a one-way ANOVA, as described in the figure legends. When more than one factor existed, a two-way ANOVA was applied with an interaction between the two factors, and, if a significant difference was found (*P*<0.05), a Tukey post-hoc test was performed.

## Results

### Stomatal anatomy

**Fig. 1.**
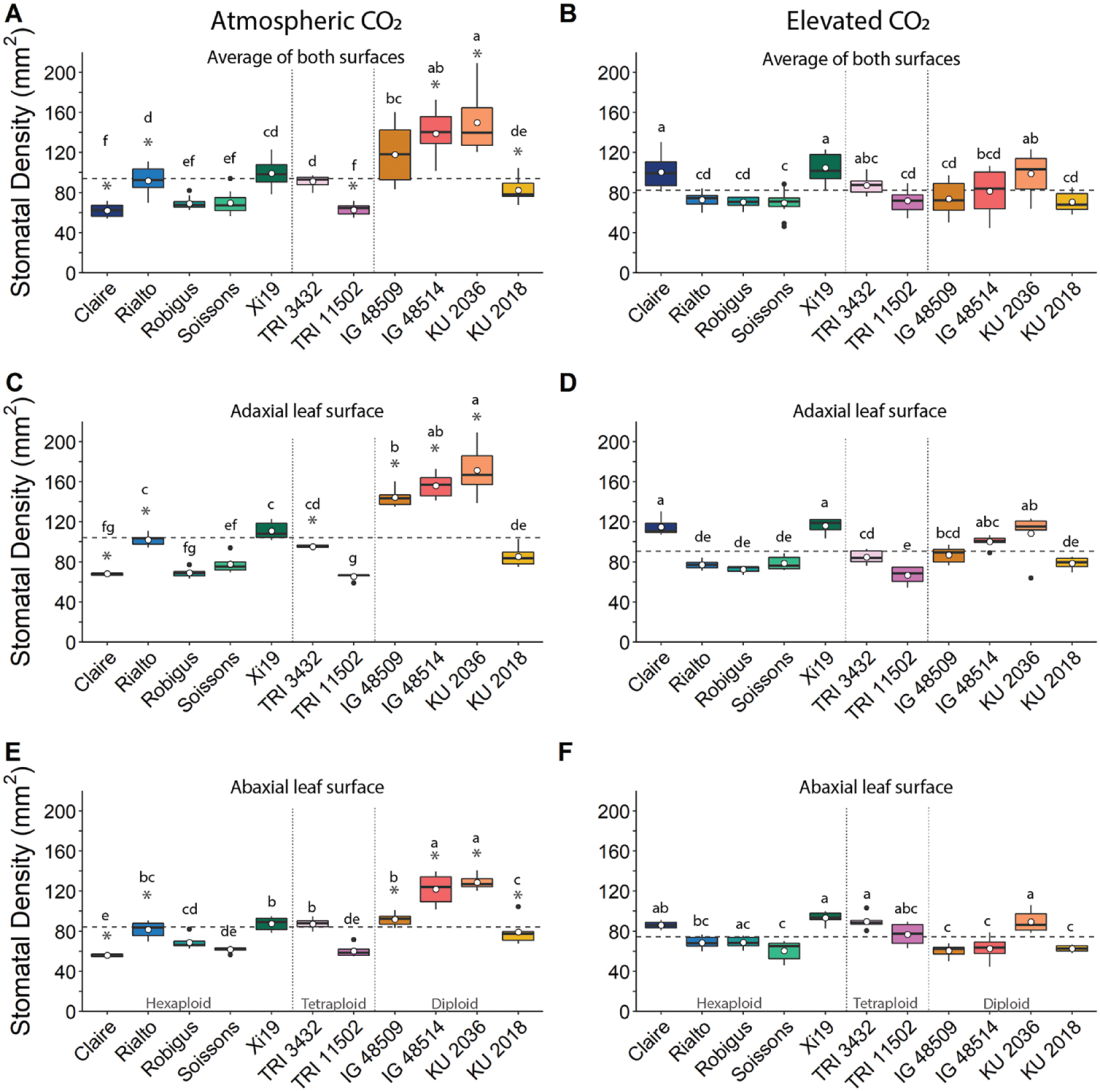
Mean (white dot) and variation (box and whisker plots displaying distribution of biological replicates) of flag leaf stomatal density (mm^2^), calculated from the average of both leaf surfaces (A and B), the adaxial leaf surface (C and D), and the abaxial leaf surface (E and F) for 11 wheat species grown at atmospheric CO2 (~408 ppm; A, C, and E) and elevated CO_2_ (~800 ppm; B, D, and F). Different letters represent statistically significant differences (*P*<0.05) between species means using the results of a Tukey test following a two-way ANOVA. A dashed line represents mean stomatal density of all wheat lines for the specific CO_2_ treatment and leaf surface. Dotted lines separate wheat by ploidy. To test the effect of growth at elevated [CO_2_] on stomatal density, a *t*-test with a Bonferroni–Hochberg end correction (*n*=6) was used to compare stomatal density means of individual wheat lines, with gray asterisks indicating significant differences (*P*<0.05).

Stomatal anatomy including SD and GCL was measured in five elite *T. aestivum* cultivars (Claire, Rialto, Robigus, Soissons, and Xi19; all hexaploid) and six WRs (four diploid lines IG 48509, IG 48514, KU 2018, and KU 2036; and two tetraploid lines TRI 3432 and TRI 11502), grown at two [CO_2_], atmospheric (a[CO_2_]) at ~408 ppm and elevated (e[CO_2_]) at ~800 ppm. Significant (*P*<0.05) variation in combined (adaxial+abaxial) leaf SD was found between species grown at a[CO_2_] (Fig. 1A) with the hexaploid cultivars ranging from ~0 mm^2^ to ~100 mm^2^ and the wheat relatives showing a larger range of ~60 mm^2^ to ~160 mm^2^, with a +60% difference between the lowest and the highest mean SDs. When grown at e[CO_2_] (Fig. 1B), less variation between and within species was observed. The majority of WRs showed a decrease in SD, with the exception of the *T. dicoccoides* accession TRI 11502 in which SD increased. No consistent pattern of change was observed for the elite hexaploid cultivars, with two cultivars showing no change in SD, while Rialto decreased, and Claire increased SD (*P*<0.05). SD was higher on the adaxial (upper) leaf surface (Fig. 1C) compared with the abaxial (lower) (Fig. 1E) surface (*P*<0.05) and SD on the adaxial surface was influenced to a greater extent by e[CO_2_] (Fig. 1D) and accounted for a greater proportion of changes in total leaf SD compared with the abaxial surface, and this was particularly evident in the WRs. Overall, there was no relationship between SD in plants grown a[CO_2_] and e[CO_2_] (Supplementary Fig. S1A). However, those species showing a change in SD with e[CO_2_] on the adaxial surface also had significantly altered SD on the abaxial surface, albeit of a smaller magnitude (Fig 1F). These data suggest that the majority of the combined (adaxial+abaxial) SD is determined by adaxial density (Fig. 1). Although there was no consistent species response of GCL to growth at e[CO_2_] (Supplementary Fig. S2), a significant (*P*=0.0055) positive correlation was observed between GCL from plants grown at ambient and elevated [CO_2_] (Supplementary Fig. S1B). The smallest GCL was observed for *Ae. tauschii* accession KU 2036 at ~29 μm at a[CO_2_] and ~35 μm at e[CO_2_], while the largest GCL was found on the bread wheat cultivars Xi19 at ~47 μm at a[CO_2_] and Robigus at ~45 μm at e[CO_2_]. When species were separated by ploidy, diploid species tended to respond to e[CO_2_] by increasing GCL; however, this was not always significant (Supplementary Fig. S2). Tetraploids had a tendency to decrease in GCL, but no specific trends were observed for hexaploids. Unlike the case for SD, it appears that average leaf GCL was determined by both the adaxial and abaxial leaf surfaces, as similar responses to e[CO_2_] were observed on both, and together reflected the observed differences in combined (adaxial+abaxial) leaf averages (Supplementary Fig. S2).

**Fig. 2.**
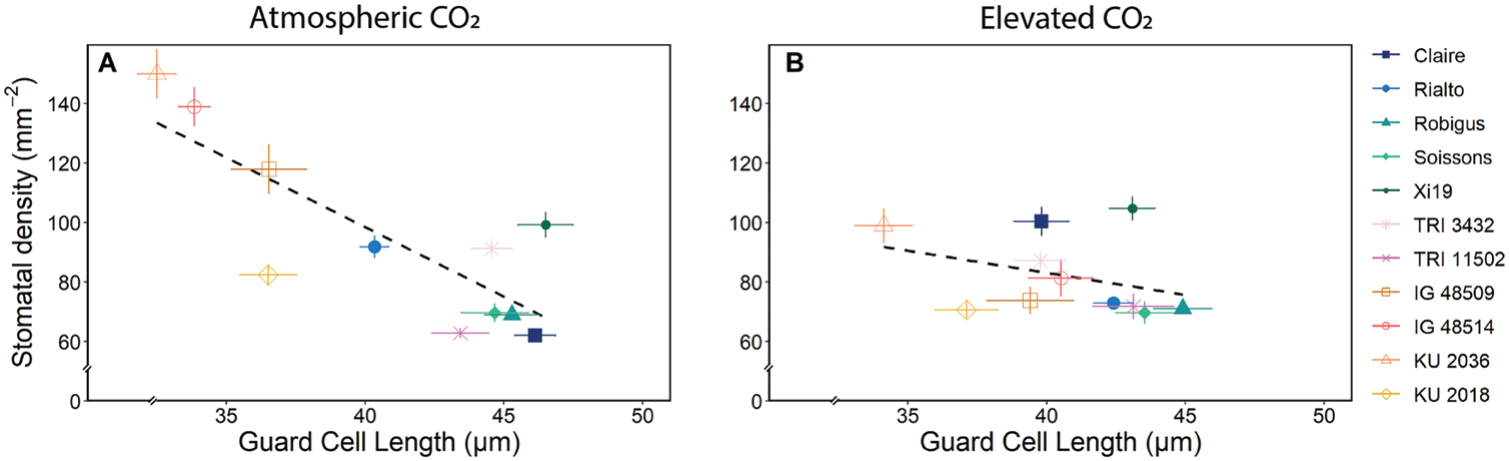
Correlation between total stomatal density (mm^2^) and total guard cell length (µm) for each species, calculated for the average of both leaf surfaces, for 11 wheat species grown at atmospheric CO_2_ (~408 ppm; A) and elevated CO_2_ (~800 ppm; B). The black dotted line represents the trend in the data between the two variables. Atmospheric CO_2_ correlation= –0.552 (*P*=6.79e-12) and elevated CO_2_ correlation=0.0625 (*P*=0.483) using a Pearson’s correlation test. Error bars represent the SE (*n*=12).

A negative correlation between SD and GCL was evident for wheat grown at ambient [CO_2_] (*P≤*0.001; Fig. 2A), demonstrating a relationship between decreasing SD and ­increasing stomatal size, driven mostly by the change in the four diploid accessions. *Aegilops tauschii* accession KU 2036 had the highest SD and smallest GCL, and the accession with the lowest SD mean (cv. Claire) had one of the largest GCLs. Although a similar trend of decreasing GCL with increasing SD was observed for the species and cultivars when grown under e[CO_2_], this relationship was not significant (Fig. 2B). This is most likely to be attributable to the reduced range in SD under e[CO_2_], particularly for the four *Ae. tauschii* accessions (Fig. 1B).

### Anatomical potential maximum rate of *g*_ s_

**Fig. 3.**
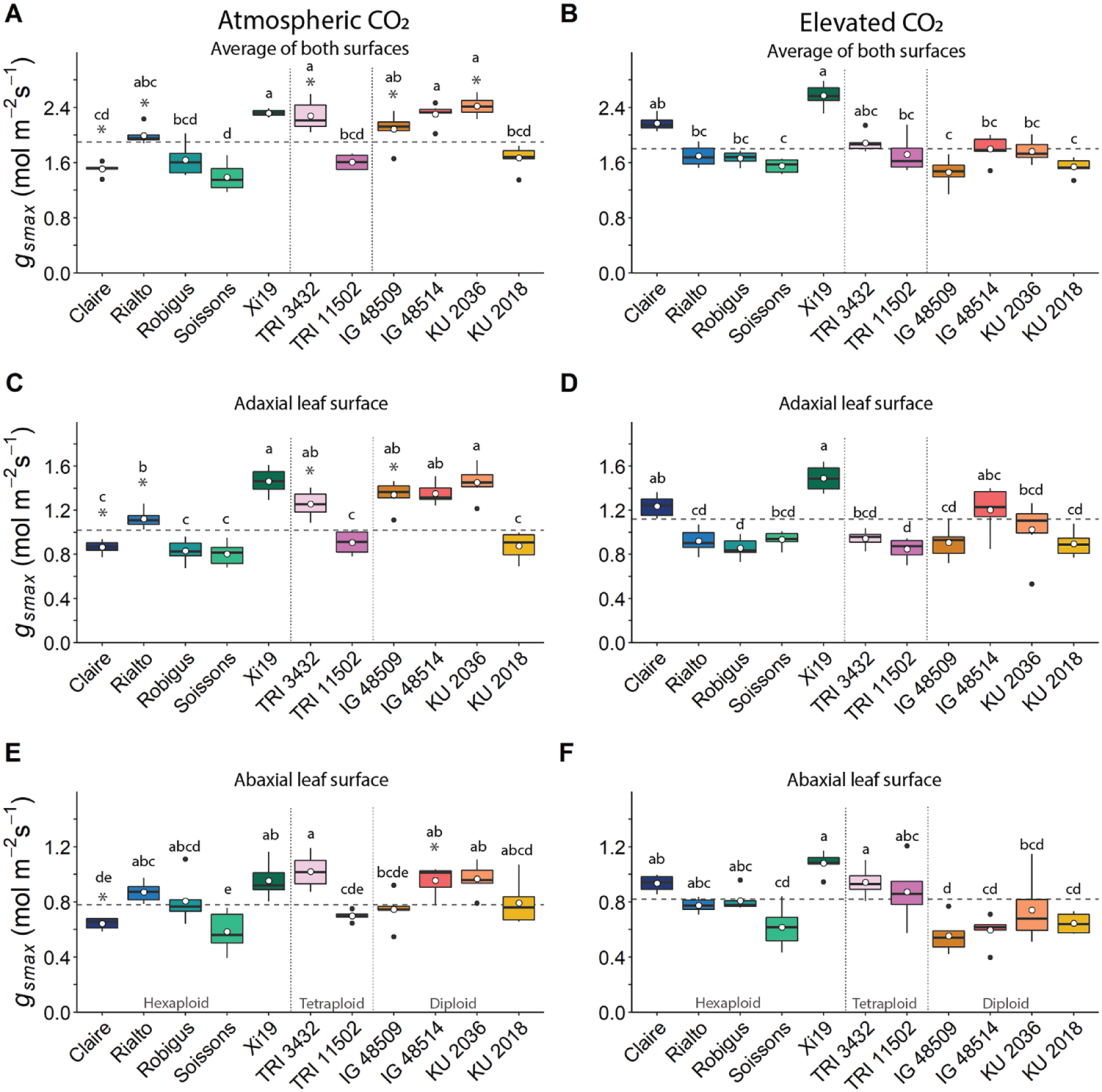
Mean (white dot) and variation (box and whisker plots displaying distribution of biological replicates) of flag leaf *g*_smax_ (mol m^–1^ s^–1^), calculated from the average of both leaf surfaces (A and B), the adaxial leaf surface (C and D), and the abaxial leaf surface (E and F) for 11 wheat species grown at atmospheric CO_2_ (~408 ppm; A, C, and E) and elevated CO_2_ (~800 ppm; B, D, and F). Different letters within each graph represent statistically significant differences (*P*<0.05) between means using the results of a Tukey test following a two-way ANOVA. The dashed line represents the mean *g*_smax_ of specific CO_2_ treatment and leaf surface. Dotted lines separate wheat by ploidy. To test the effect of growth at elevated [CO_2_] on *g*_smax_, a *t*-test with a Bonferroni–Hochberg end correction (*n*=6) was used to compare individual wheat line means, with gray asterisks indicating significant differences (*P*<0.05).

SD and measurements of stomatal size (GCL) were used to calculate the maximum anatomical stomatal conductance (*g*_smax_), assuming fully open pores. At a[CO_2_], a considerable range of *g*_smax_ values were determined between accessions (Fig. 3A), with cv. Soissons displaying the lowest and *Ae. tauschii* accession KU 2036 the highest values (an increase of ~41%, driven predominantly by the differences in SD; Fig. 1). Higher *g*_smax_ values were observed on the adaxial leaf surface irrespective of growth [CO_2_] (Fig. 3C, D), with typical values >1.0 mol m^–2^ s^–1^, whereas *g*_smax_ values on the abaxial surface (except e[CO_2_]-grown Xi19) were <1.0 mol m^–2^ s^–1^ (Fig. 3E, F). There was considerable variation in the response of *g*_*smax*_ when grown under e[CO_2_] compared with a[CO_2_] (Fig. 3B). These differences appear to be driven mostly by the changes in SD (Fig. 1), but not exclusively as the CO_2_ response patterns between SD (Fig. 1) and *g*_smax_ (Fig. 3) were not identical. Similar to the patterns described for SD, there was a tendency for reduction in *g*_smax_ with e[CO_2_] driven mostly by adaxial *g*_smax_. However, interestingly, not all of the changes in SD translated into changes in *g*_smax_, strongly indicating a role for changes in GCL with e[CO_2_] to compensate for changes in density, maintaining a similar *g*_smax_ (Lawson and Morison, 2004; Harrison *et al.*, 2020; Wall *et al.*, 2022); for example, the diploid and tetraploid species have similar *g*_smax_ to that of the hexaploid species even though SD is much higher in the diploid species.

### Leaf gas exchange

#### *Response of* g_*s*_*and* A *to a step change in PPFD*

**Fig. 4.**
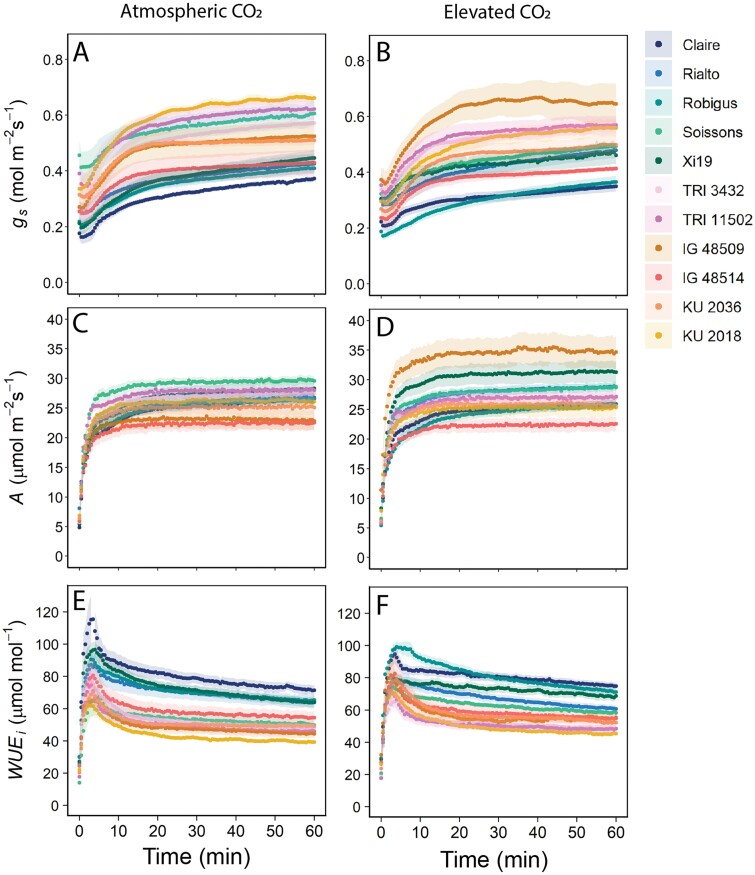
Temporal response of stomatal conductance (*g*_s_; A and B), net CO_2_ assimilation (*A*; C and D), and intrinsic water use efficiency (WUEi; E and F), to a step increase in light intensity (from 100 μmol m^–1^ s^–1^ to 1000 μmol m^–1^ s^–1^ PPFD for 60 min) for 11 wheat species grown at atmospheric CO_2_ (~408 ppm) and elevated CO_2_ (~800 ppm). Gas exchange parameters (*g*_s_ and *A*) were recorded at 30 s intervals, and leaf temperature and VPD were maintained at 22 °C, and 1 ± 0.2 kPa, respectively. Error ribbons represent the mean ±SE (*n*=5–7).

The effect of e[CO_2_] on stomatal behavior/kinetics was assessed by measurements of *g*_s_ and *A* following a step increase in PPFD (Fig. 4). As expected, all species and cultivars exhibited an increase in *g*_s_ and *A* with increasing irradiance. In general, *A* rapidly increased compared with *g*_s_ when light was increased (Fig. 4A–D), and this resulted in the maximum WUEi value being reached within a few minutes of the change in PPFD (Fig. 4E, F). Further increases in *g*_s_ with time drove a continuous decrease in WUEi, and this trend continued after *A* had reached a maximum steady state. Considerable variation in *A*, *g*_s_, and WUEi was observed in plants grown under both [CO_2_] treatments, although the variation was more apparent in growth at e[CO_2_], particularly for *A* and *g*_s_ (Fig. 4A–D).

**Fig. 5.**
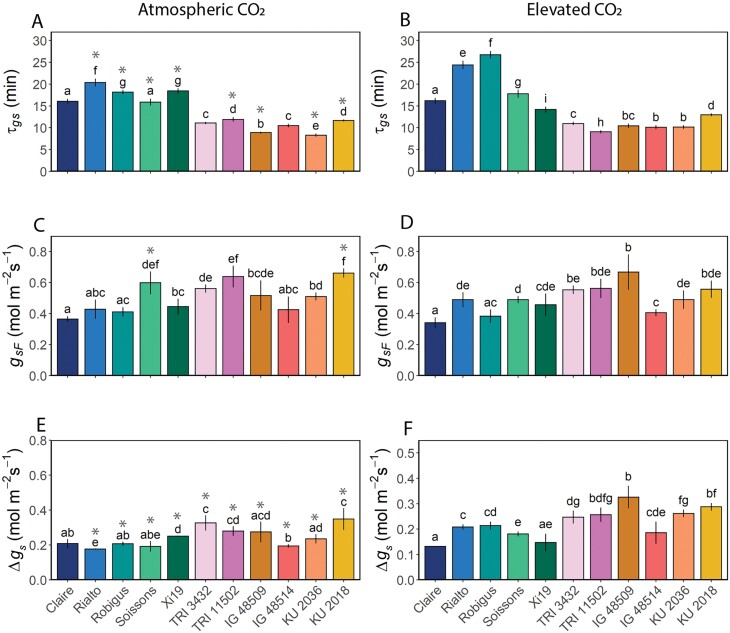
Time constant for stomatal opening [τ_*g*s_ (min); A and B], final stomatal conductance value [*g*_sF_ (mol m^–2^ s^–1^); B and D] after a step increase in light intensity from 100 μmol m^–2^ s^–1^ to 1000 μmol m^–2^ s^–1^ PPFD, and the difference in *g*_s_ [Δ_*g*s_ (mol m^–2^ s^–1^)] between 100 μmol m^–2^ s^–1^ and 1000 μmol m^–2^ s^–1^ PPFD (E and F). The 11 wheat species were grown at both at atmospheric [CO_2_] (~408 ppm; A, C, and E) and elevated [CO_2_] (~800 ppm; B, D, and F). Error bars represent 95% confidence intervals using the results of a Tukey test following a two-way ANOVA. To test the effect of growth at elevated [CO_2_], a *t*-test with a Bonferroni–Hochberg end correction (*n*=5–7) was used to compare individual wheat line means, with gray asterisks indicating significant differences (*P*<0.05).

**Fig. 6.**
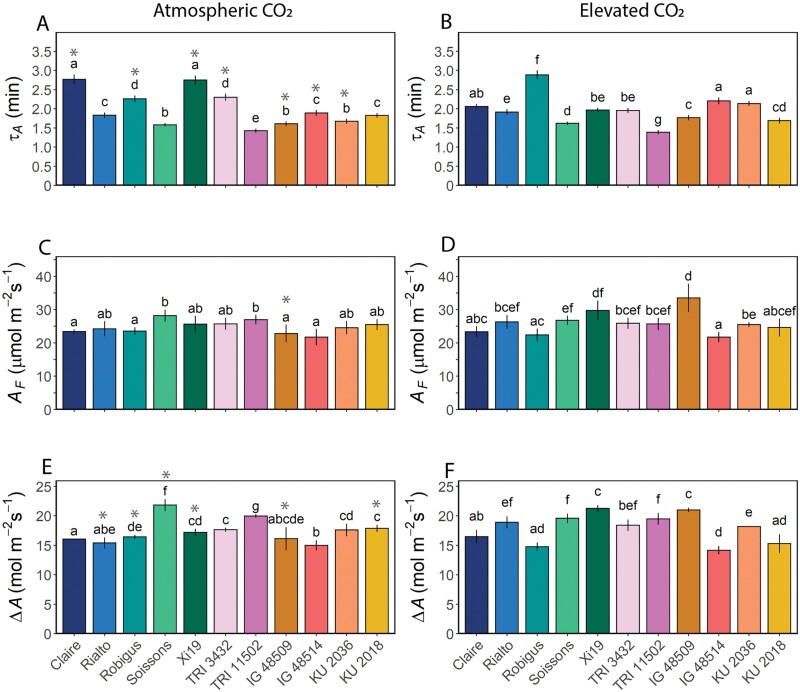
Time constant for light-saturated carbon assimilation [τ_*A*_ (min); A and B), final light-saturated carbon assimilation rate [*A*_F_ (µmol m^–2^ s^–1^); B and D] after a step increase in light intensity from 100 μmol m^–2^ s^–1^ to 1000 μmol m^–2^ s^–1^ PPFD, and the difference in *A* [*ΔA* (µmol m^–2^ s^–1^)] between 100 μmol m^–2^ s^–1^ and 1000 μmol m^–2^ s^–1^ PPFD (E and F). The 11 wheat species were grown at both at atmospheric [CO_2_] (~408 ppm; A, C, and E) and elevated [CO_2_] (~800 ppm; B, D, and F). Error bars represent 95% confidence intervals using the results of a Tukey test following a two-way ANOVA. To test the effect of growth at elevated [CO_2_], a *t*-test with a Bonferroni–Hochberg end correction (*n*=5–7) was used to compare individual wheat line means, with gray asterisks indicating significant differences (*P*<0.05).

The time constant to reach 63% of the final value for *g*_s_ (τ_*g*s_) as an indicator of the rapidity (Fig. 5) was significantly greater (*P*<0.05) in the hexaploid wheat compared with other species, regardless of growth [CO_2_] (Fig. 5A, B). Hexaploid lines averaged 20 min to reach maximum *g*_s_ while the other species averaged 10 min. In general, e[CO_2_] increased the time constant (indicating slower stomatal responses) in most species with the exception of cv. Claire and *T. dicoccon* accession TRI 3432 which showed no significant differences, and *T. dicoccoides* accession TRI 11502 and cv. Xi19 which were significantly faster with e[CO_2_] (Fig. 5B). Interestingly, there was a significant positive correlation (*P*<0.05) between τ_*g*s_ in plants grown at ambient [CO_2_] and at e[CO_2_], indicating that speed was inherent with limited influence of growth environment (Supplementary Fig. S3). However, the speed of the *g*_s_ response did not influence the overall final *g*_s_ (*g*_sF_) achieved (Fig. 5C–F), with no correlation observed between the two. Growth at e[CO_2_] did not influence *g*_sF_ values, with no differences observed between most accessions, with the exception of cv. Soissons and KU 2018 (Fig. 5C, D). On the other hand, the magnitude of change in *g*_s_ (Δ*g*_s_) decreased in almost all species and cultivars with growth at e[CO_2_], with the exception of the hexaploid cv. Rialto and Robigus and the *Ae. tauschii* accession IG 48509, and this was related to the speed of response, with slow responding accessions (mostly the hexaploids) having a lower Δ*g*_s_ compared with the fast responders in which Δ*g*_s_ was greater (Fig. 5E), although this correlation was only significant (at *P*=0.0107) when plants were grown under e[CO_2_] and not significant at ambient a[CO_2_] (Supplementary Fig. S4A, B). The fact that there was no effect of e[CO_2_] on *g*_sF_ indicates that minimum *g*_s_ must have been higher with growth at high [CO_2_]. The more rapid *g*_s_ responses did not, however, impact on τ_*A*_ (Fig. 6), with no clear relationship between the two parameters. Δ*g*_s_ was positively correlated with *g*_sF_ at both a[CO_2_] and e[CO_2_] (Supplementary Fig. S4C, D). Under both ambient and elevated growth [CO_2_] conditions, the greater the *g*_*s*F_, the higher the *A*_F_ achieved (Supplementary Fig. S4E, F), and under ambient but not elevated [CO_2_] this was also correlated with a greater change in *A* (Δ*A*) (Supplementary Fig. S4G, H), suggesting diffusion constraints by *g*_s_ on the kinetic responses of *A.* The final value of *A* (*A*_F_, Fig. 6) at 1000 μmol m^–2^ s^–1^ PPFD was similar across the different accessions, ~25 μmol m^–2^ s^-1^ for both CO_2_ growth treatments, although values for two accessions were significantly (*P*<0.05) higher when grown at e[CO_2_], hexaploid cv. Xi19, ~32 μmol m^–2^ s^–1^ and *Ae. tauschii* accession IG 48509, ~35 μmol m^–2^ s^–1^, and, not unexpectedly, this was highly positively correlated with the Δ*A* (Supplementary Fig. S4I, J). The change in *A* (Δ*A*) was similar across the different species, showing a typical increase of 15 μmol m^–2^ s^–1^ with a few exceptions being higher at 20 μmol m^–2^ s^–1^ (Fig. 6E, F). However, there was no relationship between τ_*A*_ and these values in plants grown under e[CO_2_] (Fig. 6), but the speed of the *A* response was negatively correlated with *g*_sF_, suggesting possible differences in the induction of photosynthesis due to both stomatal and biochemical constraints (Supplementary Fig. S4K, L).

#### *A/C*_ i_ response analysis

In order to assess changes to photosynthetic capacity, the response of assimilation rate (*A*) as a function of internal [CO_2_] (*C*_i_; Supplementary Fig. S5) was determined on the flag leaf on plants grown in the two [CO_2_] environments. All accessions exhibited the expected increase in *A* with increased *C*_i_ before reaching a plateau. Accessions grown at ambient [CO_2_] displayed significant variation in their responses (Supplementary Fig. S5A). In general, hexaploid accessions had the highest assimilation rates, and greater *V*_cmax_, *J*_max_, and *A*_max_ values at both ambient and e[CO_2_] (Supplementary Fig. S6) whilst those of the tetraploid and diploid species were lower, indicating a reduced photosynthetic capacity. Growth under e[CO_2_] had no significant influence on photosynthetic capacity, in any of the species.

#### Plant growth

Multiple leaf growth parameters were measured including flag leaf area (LA; Supplementary Fig. S7), DW (Supplementary Fig. S8), and leaf thickness (LT; Supplementary Fig. S8). In general, all hexaploid wheat accessions had a greater LA (Supplementary Fig. S7) than other species, except for the tetraploid *T. dicoccon* accession TRI 3432 when grown at a[CO_2_]. A similar trend followed for e[CO_2_]-grown wheat, although there was less variation between species. No significant differences were observed between accessions from the same species from a[CO_2_] to e[CO_2_] except for *Ae. tauschii* accession IG 48509 in which LA increased. DW (Supplementary Fig. S8) followed the same trends as LA. In general, there was a trend for hexaploid and tetraploid species having thicker leaves than the diploid species at both CO_2_ growth treatments (Supplementary Fig. S9), the exception being cv. Soissons in which LT was reduced at e[CO_2_]. These data suggest that the diploid species had smaller thinner leaves compared with the hexaploid wheat species.

## Discussion

The global human population is expected to reach >9.5 billion by 2050, putting increasing pressure on breeders and crop scientists to improve yields to ensure sufficient food (Asseng *et al.*, 2020). However, with the continued increases in global [CO_2_], along with predicted changes to climate, it is vital that crop improvement programs consider the impact of these changes on crop performance and identify valuable physiological resilience traits (and the underlying genetics) that maintain productivity in a diverse range of environmental conditions. Genetic engineering approaches have demonstrated that enhancing photosynthetic capacity and stomatal behavior can successfully deliver crops with greater yield and resource use efficiency (Ruiz-Vera *et al.*, 2017; López-Calcagno *et al.*, 2020; De Souza *et al.*, 2022). However, another powerful approach is exploiting natural variation in various physiological traits including photosynthesis (Driever *et al.*, 2014; Carmo-Silva *et al.*, 2017; Faralli and Lawson, 2019) and stomatal dynamics (Faralli *et al.*, 2019, 2022; Sakoda *et al.*, 2022). Exploiting variation in current elite bread wheat germplasm (e.g. Driever *et al.*, 2014; Faralli *et al.*, 2019) as well as crop relatives (McAusland *et al.*, 2020; Sharwood *et al.*, 2022) offers significant potential to identify novel allelic variation (Sakoda *et al.*, 2022; Sharwood *et al.*, 2022;  Yin *et al.*, 2022). Here we have explored the impact of growth [CO_2_] on variation in photosynthesis, stomatal anatomy, and stomatal kinetics in several elite wheat cultivars and their tetraploid and diploid relatives.

It is well documented that significant variation in stomatal anatomy exists between and within species, spatially within leaves (Ticha, 1982; Smith *et al.*, 1989; Willmer and Fricker, 1996; Weyers and Lawson, 1997; Weyers *et al.*, 1997) and on different leaf surfaces (Wall *et al.*, 2022), all of which are influenced by the growth environment (Poole *et al.*, 1996; Croxdale, 2000; Lawson *et al.*, 2002). Stomatal density is one of the most plastic traits and is affected by a great number of environmental parameters (Matthews and Lawson, 2019; Stevens *et al.*, 2021). Increasing growth [CO_2_] most commonly decreases SD in the majority of plant species investigated (Woodward, 1987), but not all (Lodge *et al.*, 2001), and the degree of change is not the same even within cultivars of the same species (Dusenge *et al.*, 2019). Not unexpectedly, in this study we observed significant variation across and between species and cultivars. The highest SDs were observed in the tetraploid relatives, with some individuals having double that of some elite varieties. A possible explanation for the high SD in the WRs is the smaller leaf area in these species (Supplementary Fig. S7). Therefore, expansion or differentiation of the epidermal cells in the elite cultivars would reduce SD (Lawson *et al.*, 2002). Interestingly, growth at e[CO_2_] generally reduced SD in diploid species, but not in the elite cultivars (except Rialto), and therefore no relationship between SD in plants grown under the two [CO_2_] was observed (Supplementary Fig. S1). Furthermore, variation within and between cultivars was generally reduced at e[CO_2_], although the underlying cause of the reduced variation is currently unknown. However, as these plants were grown in controlled environments, and the only changing variable was [CO_2_] (with all other parameters kept constant), it is possible that plants grown at a[CO_2_] were subjected to greater variation in [CO_2_] (due to photosynthetic draw down), and that the plants were more sensitive and responsive to this variation. For example, the a[CO_2_] growth chambers ware maintained at 400 ppm; however, photosynthetic CO_2_ fixation would result in short-term dynamic draw down of [CO_2_] to ~320 ppm, whilst the same draw down in e[CO_2_] would result in variation only between 700 ppm and 800 ppm, and plants would be less sensitive to these changes (Franks *et al.*, 2012) as these levels will saturate photosynthesis (Supplementary Figs S2, S3).

The SD variation and response to [CO_2_] were mainly the result of anatomical changes on the adaxial leaf surface, suggesting two important points. Firstly, the receptors or signaling pathways responsible for detecting and responding to growth at e[CO_2_] which drive changes in stomatal patterning are complex and either they reside separately on the two surfaces (and are not mesophyll driven) or there is limited surface to surface communication. Secondly, stomata on the adaxial surface play a more prominent role in gaseous exchange than those on the abaxial surface. This agrees with the recent work by Wall *et al.* (2022) who demonstrated that adaxial stomata make the greatest contribution to leaf gas exchange in amphistomatous bread wheat. Tsutsumi *et al.* (2014) reported that elevated [CO_2_] decreased leaf size in rice, and this was accompanied by a decrease in epidermal cell numbers on the adaxial surface, but a reduction in cell size on the abaxial surface, thus providing a possible explanation for the differences observed between surfaces in different cultivars. GCL (as an indicator of stomatal size) was generally lower in the WRs compared with the elite cultivars, and together with SD was used to determine the maximum potential *g*_s_ (*g*_smax_) for the accessions investigated. As above, the variation in *g*_smax_ was driven mostly by SD (and at the leaf level due to differences on the adaxial surface), but not entirely, with GCL clearly having a secondary role, as has previously been shown (Lawson and Morison, 2004). These findings indicate that there are some compensatory processes between SD and GCL (or size) to maintain a level of *g*_smax_ across species (Lawson and Morison, 2004; Bussis *et al.*, 2006; McElwain *et al.*, 2016). The strong negative correlation observed between SD and stomatal size in plants grown at ambient [CO_2_] agrees with several reports that have shown that lowered SD results in increased size (Franks and Farquhar, 2007). What is particularly interesting is that this size–density relationship was lost in plants grown at e[CO_2_], due in part to the decrease in SD variation with growth at e[CO_2_], and the fact that no relationship between SD at the two growth [CO_2_] were observed; however, GCL was positively correlated between plants grown in the two environments. The anatomical constraints of *g*_smax_ can translate into species-specific differences in operational or functional *g*_s_ (McElwain *et al.*, 2016), often with implications for carbon gain and water use efficiency—particularly in dynamic environments (Vialet-Chabrand *et al.*, 2016; Lawson and Vialet Chabrand, 2019). Dynamic stomatal responses and the speeds of stomatal responses to changing environmental cues have recently received considerable attention for optimizing *A* relative to water loss and WUEi (Farquhar and Sharkey, 1982; Mansfield *et al.*, 1990; Lawson *et al.*, 2010; Buckley and Mott, 2013; Lawson and Blatt, 2014; Buckley, 2017; Vialet-Chabrand *et al.*, 2017; Matthews *et al.*, 2018; Papanatsiou *et al.*, 2019; Yamori *et al.*, 2020). Stomatal conductance, although closely correlated with *A*, is an order of magnitude slower to respond to these changes than photosynthetic responses and can therefore lead to a disconnect between *A* and *g*_s_, as slow stomatal opening can limit CO_2_ uptake whilst slow closure can erode water use efficiency (Drake *et al.*, 2013; Lawson and Vialet-Chabrand, 2019; Vialet-Chabrand and Lawson, 2019). Exploiting variation in kinetic stomatal responses has been proposed as a possible route to increase the speed of stomatal responses to be more in tune with photosynthetic demands for CO_2_ (Lawson *et al.*, 2018). We know that stomatal kinetics depend on species (McAusland *et al.*, 2016), cultivar (McAusland *et al.*, 2020; Stevens *et al.*, 2021), environmental conditions (Ainsworth and Long, 2005, 2021; De Souza *et al.*, 2020), and time of day (Matthews *et al.*, 2017). Here the kinetic responses of *g*_s_ to increasing PPFD were up to 50% slower in the elite cultivars compared with the diploid and tetraploid WRs, and growth at e[CO_2_] decreased the speed even further. This agrees with previous reports that *g*_s_ responses are slower in species with a lower density of larger guard cells (e.g. Elliott-Kingston *et al.*, 2016) as we have observed here in the WRs, and growth under elevated [CO_2_] could amplify this, dampening the *g*_s_ response (Knapp *et al.*, 1994). Surprisingly, these differences did not directly translate into differences in final *g*_s_ values at high PPFD, most probably due to greater variation in τ_*g*s_ than *g*_sF_. However, slow *g*_s_ responses did result in a lower ∆*g*_s_ under e[CO_2_], implying that stomatal speed influences overall *g*_s_ behavior at elevated but not a[CO_2_]. This is most likely to be due to a greater variation in *g*_s_ in plants grown under e[CO_2_]. ∆*g*_s_ was positively correlated with *g*_sF_ (Supplementary Fig. S4), further supporting the idea that stomatal kinetics influence overall *g*_s_ behavior and final values achieved. Such a relationship has previously been shown for tobacco, with the greater the change the higher the *g*_s_ value achieved (von Caemmerer *et al.*, 2004). Together, these findings indicate that both anatomical and biochemical/physiological components determine the speed of *g*_s_ responses (Lawson and Blatt, 2014) and that both the rapidity in stomatal responses and the magnitude of change influence *g*_s_ values. Growth under e[CO_2_] reduced the magnitude of change in *g*_s_ following the step increase in PPFD; however, it is clear that this was driven by differences in minimum *g*_s_ and not the maximum achieved (*g*_sF_). This could be due to differences in SD with growth under e[CO_2_] or that guard cell sensitivity to [CO_2_] was reduced under these conditions, ultimately increasing *g*_*s*_ at low light (Hetherington and Woodward, 2003; Chater *et al.*, 2015). The final *g*_s_ values (*g*_sF_) positively correlated with *A*_F_ at ambient and elevated [CO_2_] (Supplementary Fig. S4), clearly demonstrating a diffusional constraint on photosynthetic induction rates, and highlights the importance of stomatal behavior in carbon assimilation (Lawson *et al.*, 2012; De Souza *et al.*, 2020; Long *et al.*, 2022). *g*_sF_ also positively correlated with ∆*A*, providing further support for a diffusional constraint on *A*. The fact that a similar relationship was not observed in plants grown at e[CO_2_] is most probably due to *g*_sF_ not being influenced by growth at e[CO_2_] and therefore decreased *g*_s_ control on CO_2_ diffusion and *A*. The negatively correlation between *g*_sF_ and τ_*A*_ (at both growth [CO_2_]) illustrates the importance of *g*_s_ in photosynthetic induction (Lawson *et al.*, 2010; Long *et al.*, 2022). Furthermore, the tight correlation between ∆*A* and *A*_F_ (Supplementary Fig. S4) suggests that photosynthetic capacity at low PPFD was less variable than at high PPFD, and the final *A* reached depends on the magnitude and kinetic changes in *A*, that are driven by both stomatal and biochemical traits (Lawson *et al.*, 2012).

The kinetic responses revealed more variation between accessions in both *A* and *g*_s_ at e[CO_2_] compared with ambient; however, the two compensated for changes in one relative to the other to maintain a similar WUEi to plants grown in ambient conditions. This demonstrates the importance of measuring both physiological components that make up WUEi as well as the attributing anatomical features (Lawson *et al.*, 2010). The strong correlation between the speed of *g*_s_ at ambient [CO_2_] and e[CO_2_] indicates that the rapidity of *g*_s_ depends on stomatal anatomy and biochemistry, and not only differences in photosynthetic biochemistry. This is also supported by the lack of any influence that growth [CO_2_] had on *V*_cmax_, *A*_max_, and *J*_max_ (Supplementary Fig. S6). Therefore, stomatal speed is an inherent trait within these species and cultivars, supporting the notion that such a phenotype could be a key trait which could be incorporated for future breeding programmes.

In conclusion, this study has demonstrated that there is significant variation between species and cultivars in stomatal anatomy and function as well as photosynthetic capacity, and that growth at e[CO_2_] does not necessarily impact on all of them, or in the same way. Current hexaploid bread wheat has a number of desirable traits, such as larger leaves and higher photosynthetic capacity, lower SD with a small Δ*g*_s_ (and therefore potential water saving capacity) compared with their WRs. Furthermore, SD in these species was not influenced by growth at e[CO_2_]. It is possible that these traits have been unintentionally selected for during the breeding process. However, the WRs have much faster stomatal kinetics compared with modern wheat species, and although here this did not directly translate into improved *A* (as in previous studies) it was directly related to *g*_sF_ which correlated significantly with *A*_max_ (Supplementary Fig. S4), suggesting some reduced stomatal diffusional constraints on *A* in cultivars with greater Δ*g*_s_. Such phenotyping traits could also be beneficial for increased WUEi as well as maintaining optimal leaf temperatures, highlighting the potential to exploit natural variation in different species, WRs, and elite crop varieties to develop idiotypes to maintain productivity in future climates.

## Supplementary data

The following supplementary data are available at *JXB* online.

Fig. S1. Correlation between SD and GCL of 11 wheat species grown at a[CO_2_] and e[CO_2_].

Fig. S2. Variation of flag leaf guard cell length of both abaxial and adaxial leaf surfaces for 11 wheat species grown at a[CO_2_] and e[CO_2_].

Fig. S3. Correlations between the time constant for stomatal opening of wheat species grown at a[CO_2_] and e[CO_2_].

Fig. S4. Correlation between kinetic parameters for 11 wheat species grown at a[CO_2_] and e[CO_2_].

Fig. S5. The response of net CO_2_ assimilation to intercellular [CO_2_] under saturating PPFD for 11 wheat species grown at a[CO_2_] and e[CO_2_].

Fig. S6. Photosynthetic capacity including the maximum RuBP-saturated rate of carboxylation, the maximum RuBP-saturated rate of carboxylation, and the light- and CO_2_-saturated rate of photosynthesis for 11 wheat species grown at a[CO_2_] and e[CO_2_].

Fig. S7. Variation of flag leaf area for 11 wheat species grown at a[CO_2_] and e[CO_2_].

Fig. S8. Variation of flag leaf dry weight for 11 wheat species grown at a[CO_2_] and e[CO_2_].

Fig. S9. Variation of flag leaf thickness for 11 wheat species grown at a[CO_2_] and e[CO_2_].

erad011_suppl_Supplementary_Material

## Data Availability

The data that support the findings of this study are openly available from this link: http://researchdata.essex.ac.uk/165/
