# Supplementary material for: The impact of growth at elevated [CO2] on stomatal anatomy and behavior differs between wheat species and cultivars
Source: J Exp Bot. 2023 Jan 12;74(9):2860–74. doi: 10.1093/jxb/erad011 (PMC10134898; doi:10.1093/jxb/erad011)
Supplement: erad011_suppl_Supplementary_Material [file erad011_suppl_supplementary_material.pdf]

Supplementary Figures

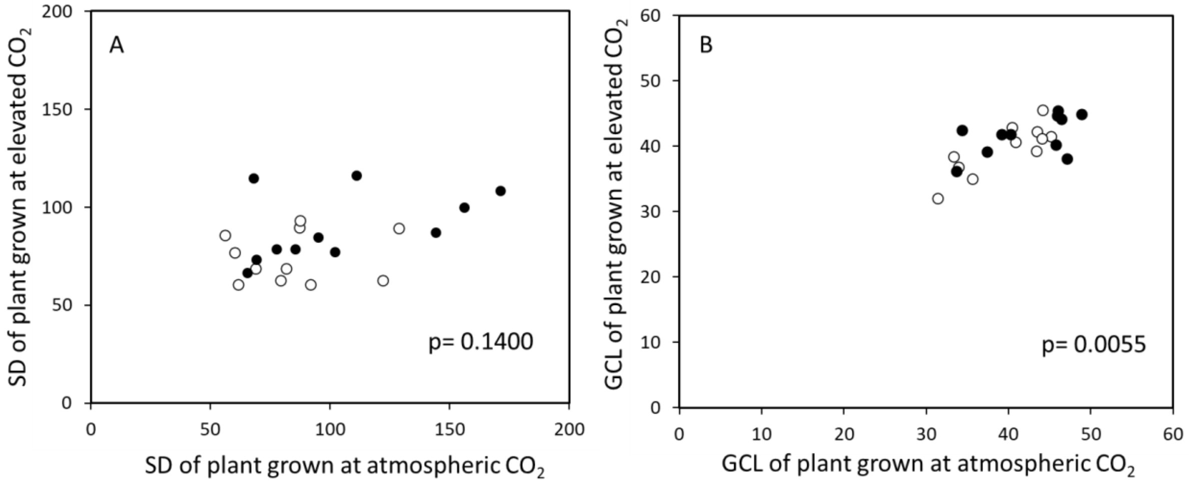

Supplementary Figure 1. Correlations between (A) averaged (n = 5-11) SD of for 11 wheat species/cultivar grown at atmospheric CO_2 and elevated CO_2 and (B) averaged CGL of for 11 wheat species/cultivar grown at atmospheric CO_2 and elevated CO_2. Open circles represent AB SD and closed circle AD SD. P value from t test (n = 11).

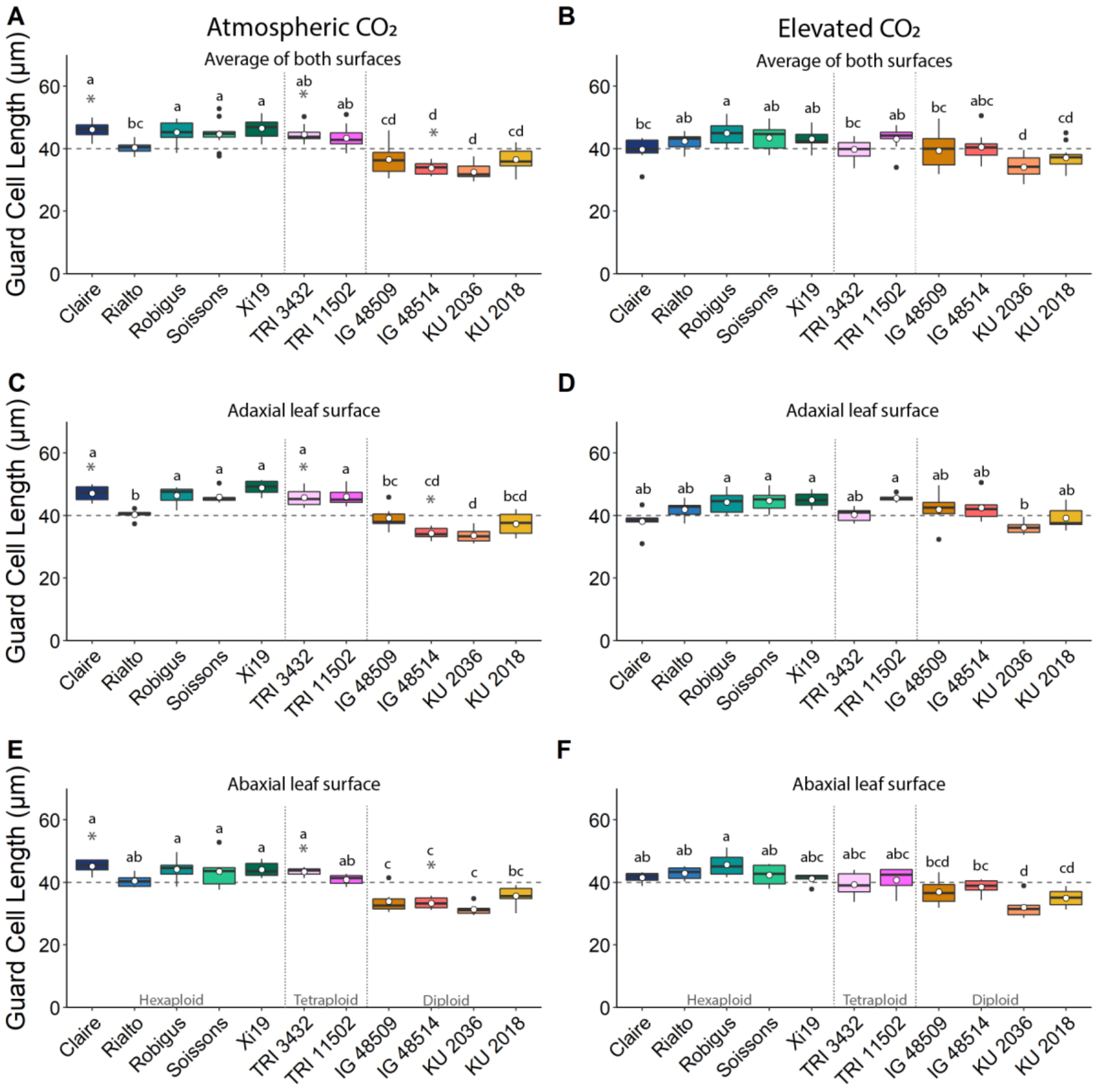

Supplementary Figure 2. Mean (white dot) and variation (box and whisker plots displaying distribution of biological replicates) of flag leaf guard cell length (µm), calculated from the mean of both leaf surfaces (A and B), adaxial (C and D) and abaxial (E and F) leaf surfaces for 11 wheat species grown at atmospheric CO_2 (~ 408 ppm; A, C and E) and elevated CO_2 (~ 800 ppm; B, D and F). Different letters within each graph represent statistically significant differences ($P < 0.05$) between means using the results of a Tukey test following a two-way ANOVA. Dashed line represents mean guard cell length of specific CO_2 treatment and leaf surface. Dotted lines separate wheat by ploidy. To test the effect of growth at elevated [CO_2] on guard cell length, a t-test with a Bonferroni-Hochberg end correction (n = 6) was used to compare individual wheat line means, with grey stars indicating significant differences ($P < 0.05$).

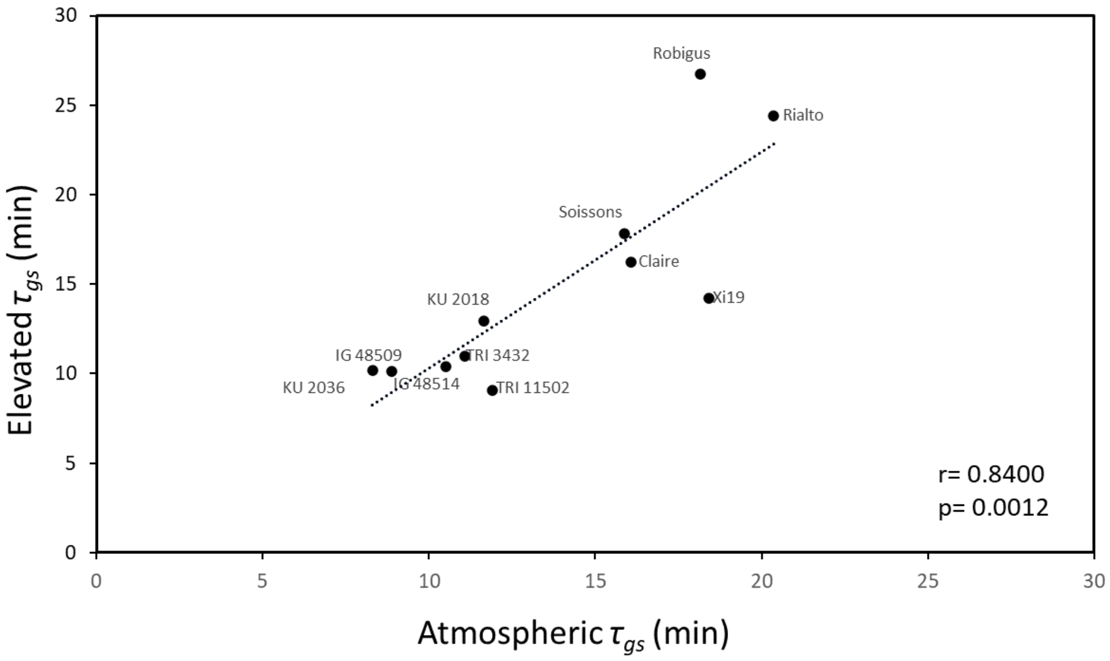

Supplementary Figure 3. Correlations between Time constant for stomatal opening (τ_{gs} (min)) for each species, for 11 wheat species/cultivar grown at atmospheric CO_2 (~ 408 ppm; A, B & C) and elevated CO_2 (~ 800 ppm; B, C & D). Black dotted line represents the trend between the two variables. Pearson's correlation r coefficient and p value from t test (n = 5-7).

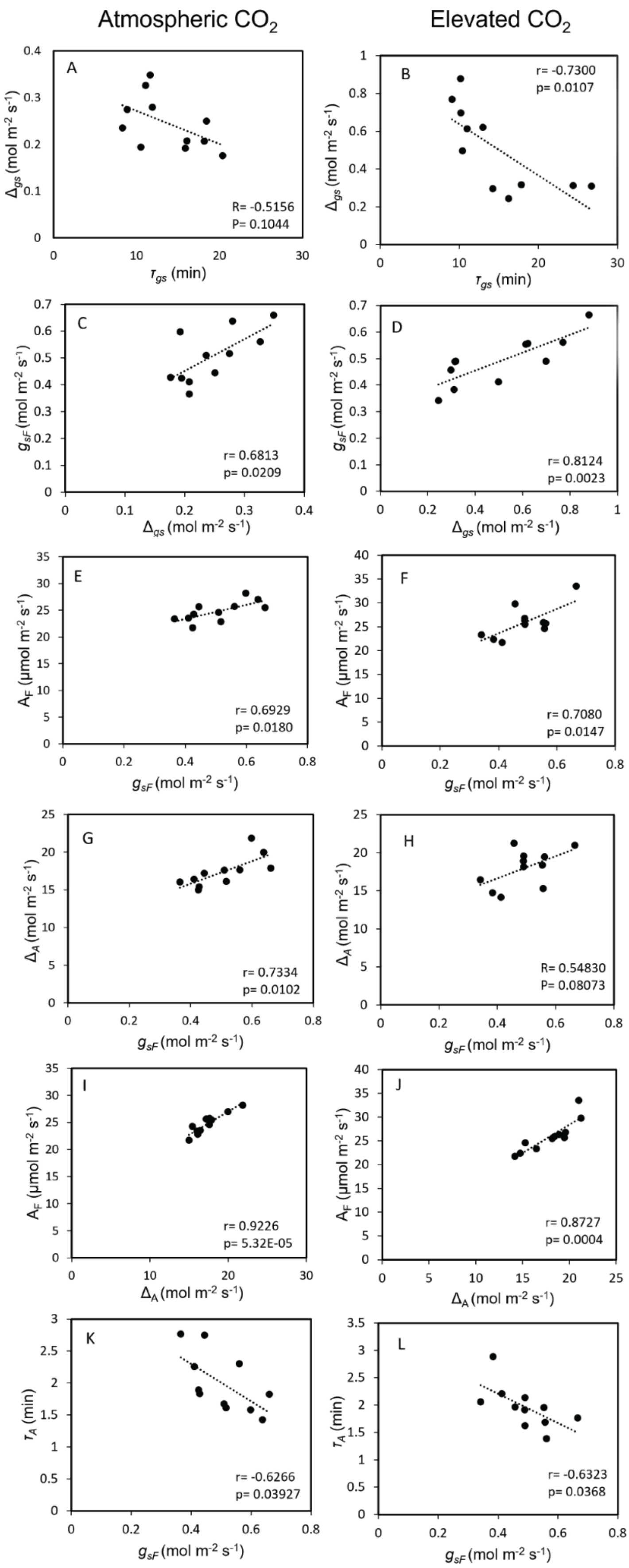

Atmospheric CO2
Elevated CO2
A
R= -0.5156
P= 0.1044
B
r= -0.7300
p= 0.0107
C
r= 0.6813
p= 0.0209
D
r= 0.8124
p= 0.0023
E
r= 0.6929
p= 0.0180
F
r= 0.7080
p= 0.0147
G
r= 0.7334
p= 0.0102
H
R= 0.54830
P= 0.08073
I
r= 0.9226
p= 5.32E-05
J
r= 0.8727
p= 0.0004
K
r= -0.6266
p= 0.03927
L
r= -0.6323
p= 0.0368
Δgs (mol m-2 s-1)
τgs (min)
gsF (mol m-2 s-1)
AF (μmol m-2 s-1)
ΔA (mol m-2 s-1)
τA (min)

Supplementary Figure 4. Correlation between 11 wheat species/cultivars grown at atmospheric CO_2 (~ 408 ppm; A, C, E, G, I) and elevated CO_2 (~ 800 ppm; B, D, F, H, J,). Time constant for light saturated carbon assimilation (τ_A (mins)), final light saturated carbon assimilation rate (A_F (μmol m^{-2} s^{-1})) after a step increase in light intensity from 100 to 1000 μmol m^{-2} s^{-1} PPFD, the difference in A (ΔA (μmol m^{-2} s^{-1})) between 100 and 1000 μmol m^{-2} s^{-1} PPFD, time constant for stomatal opening (τ_{gs} (mins)), final stomatal conductance value (g_{sF} (mol m^{-2} s^{-1})) after a step increase in light intensity from 100 to 1000 μmol m^{-2} s^{-1} PPFD and the difference in g_s (Δ_{gs} (mol m^{-2} s^{-1})) between 100 and 1000 μmol m^{-2} s^{-1} PPFD. Black dotted line represents the trend in the data between the two variables. Pearson's correlation r coefficient and p value from t test (n = 5-7).

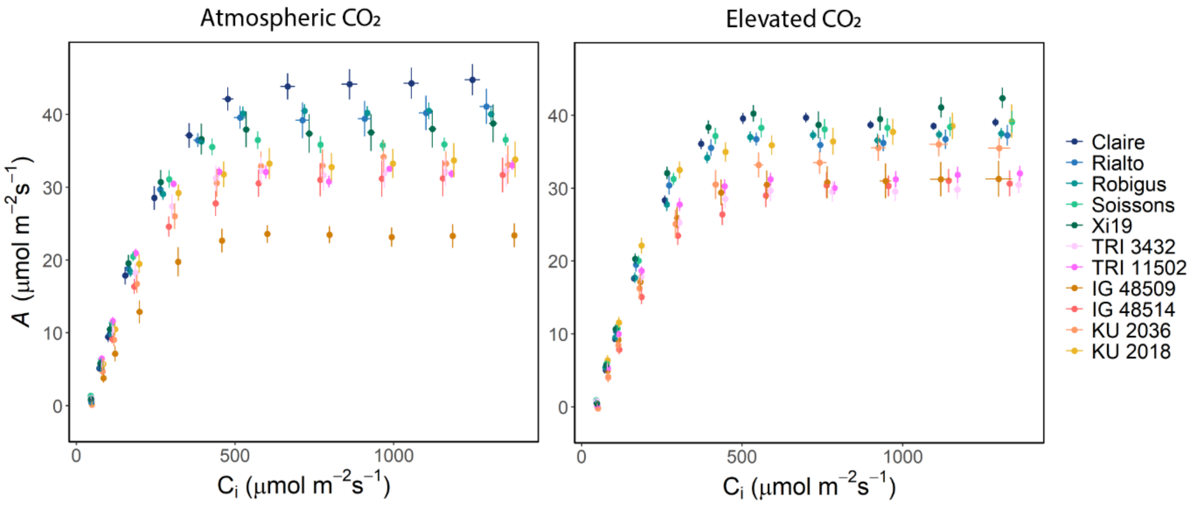

Supplementary Figure 5. The response of net CO_2 assimilation (*A*) to intercellular [CO_2] (*Ci*) between 50 and 1500 µmol m^{-2} s^{-1}, under saturating PPFD (1500 µmol m^{-2} s^{-1}), for 11 wheat species grown at atmospheric CO_2 (~ 408 ppm; A) and elevated CO_2 (~ 800 ppm; B). Error bars represent mean ± SE (n = 5-7).

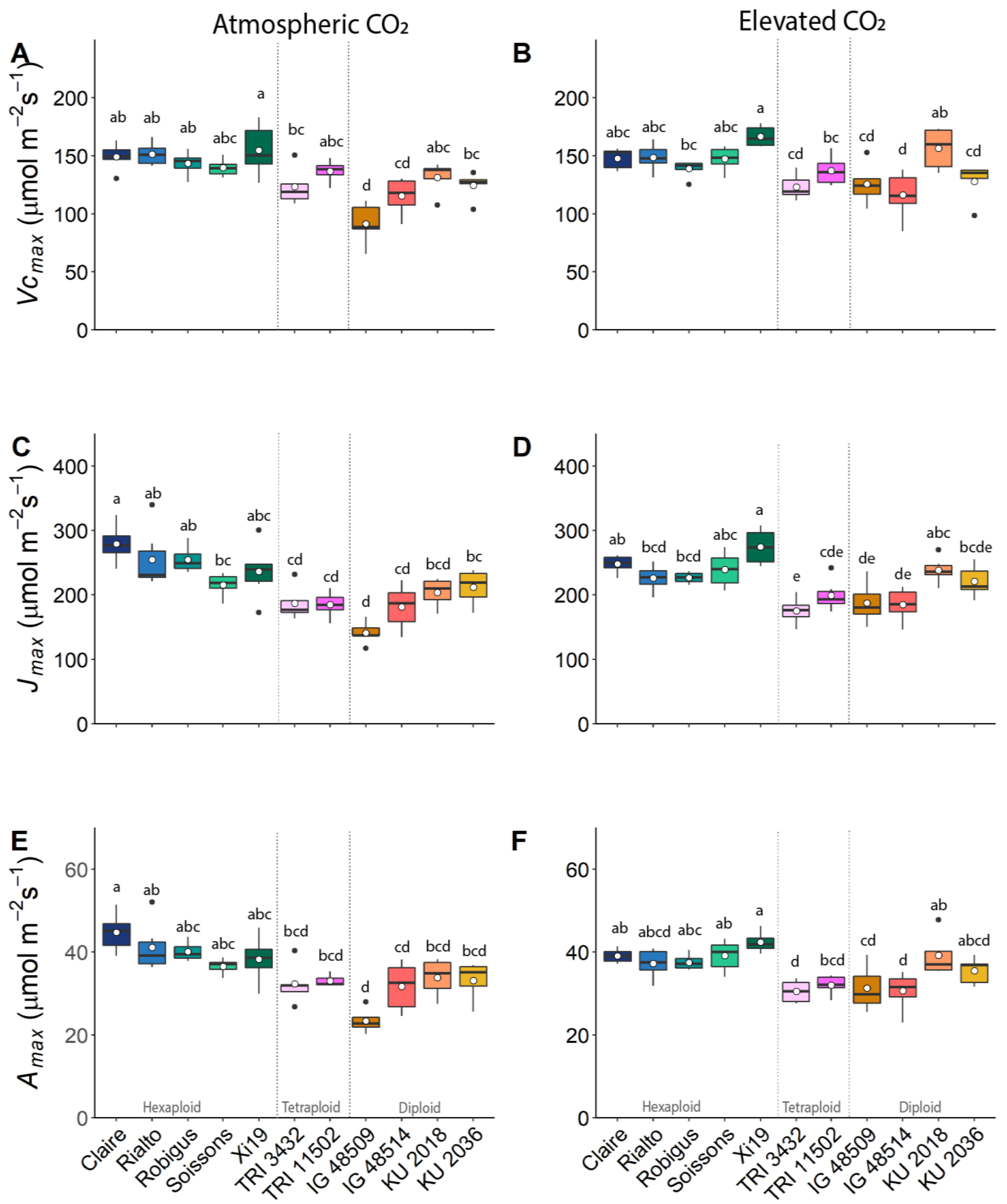

Supplementary Figure 6. Photosynthetic capacity, derived from A/C_i response of the 11 wheat species grown at atmospheric CO_2 (~408 ppm; A, C and E) and elevated CO_2 (~800 ppm; B, D and F). The maximum RuBP saturated rate of carboxylation ($V_{c,max}$ (µmol m^{-2} s^{-1}); A & B), the rate of RuBP regeneration ($J_{,max}$ (µmol m^{-2} s^{-1}); C & D) and the light and CO_2 saturated rate of photosynthesis ($A_{,max}$ (µmol m^{-2} s^{-1}); E and F). Dotted lines separate wheat by ploidy. Different letters within each graph represent statistically significant differences ($P < 0.05$) between means using the results of a Tukey test following a two-way ANOVA.

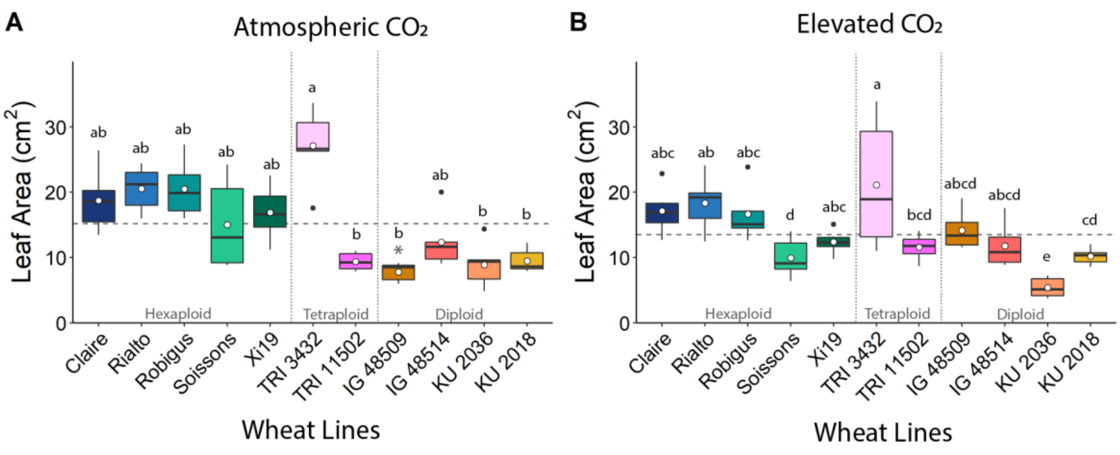

Supplementary Figure 7. Mean (white dot) and variation (box and whisker plots displaying distribution of biological replicates) of flag leaf area (cm^2) for 11 wheat species grown at atmospheric CO_2 (~ 408 ppm; A) and elevated CO_2 (~ 800 ppm; B). Different letters within each graph represent statistically significant differences ($P < 0.05$) between means using the results of a Tukey test following a two-way ANOVA. Dashed line represents mean leaf area of specific CO_2 treatment and leaf surface. Dotted lines separate wheat by ploidy. To test the effect of growth at elevated [CO_2] on leaf area, a t-test with a Bonferroni-Hochberg end correction (n = 6) was used to compare individual wheat line means, with grey stars indicating significant differences ($P < 0.05$).

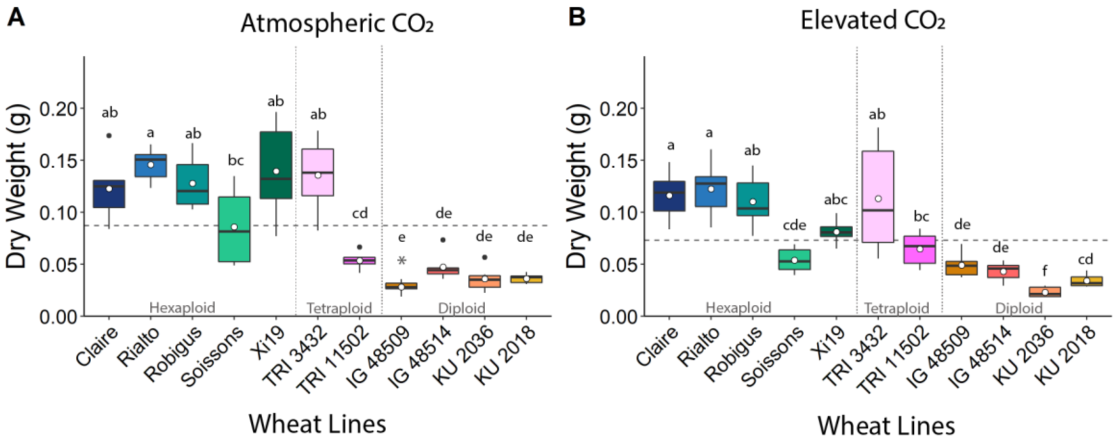

Supplementary Figure 8. Mean (white dot) and variation (box and whisker plots displaying distribution of biological replicates) of flag leaf dry weight (g) for 11 wheat species grown at atmospheric CO_2 (~ 408 ppm; A) and elevated CO_2 (~ 800 ppm; B). Different letters within each graph represent statistically significant differences ($P < 0.05$) between means using the results of a Tukey test following a two-way ANOVA. Dashed line represents mean dry weight of specific CO_2 treatment and leaf surface. Dotted lines separate wheat by ploidy. To test the effect of growth at elevated [CO_2] on dry weight, a t-test with a Bonferroni-Hochberg end correction (n = 6) was used to compare individual wheat line means, with grey stars indicating significant differences ($P < 0.05$).

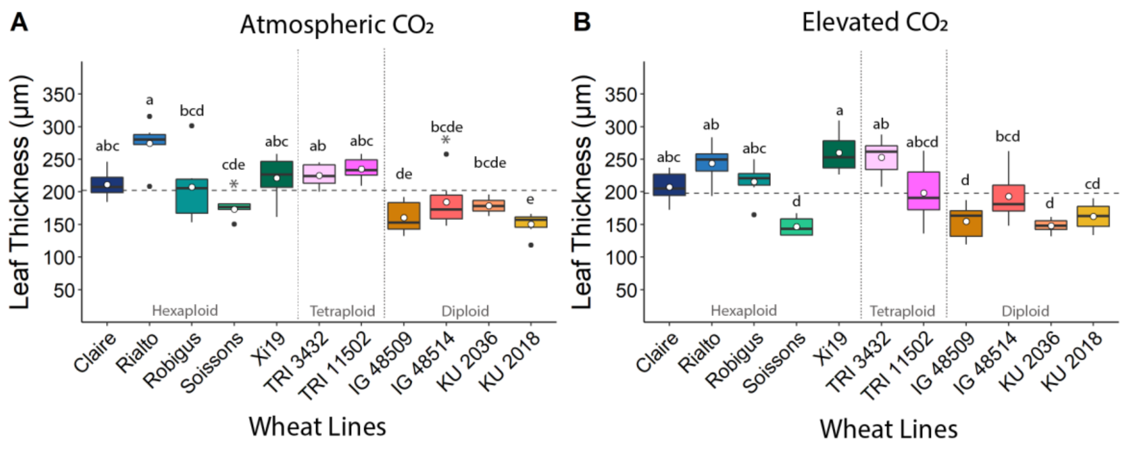

Supplementary Figure 9. Mean (white dot) and variation (box and whisker plots displaying distribution of biological replicates) of flag leaf thickness (µm) for 11 wheat species grown at atmospheric CO_2 (~ 408 ppm; A) and elevated CO_2 (~ 800 ppm; B). Different letters within each graph represent statistically significant differences ($P < 0.05$) between means using the results of a Tukey test following a two-way ANOVA. Dashed line represents mean leaf thickness of specific CO_2 treatment and leaf surface. Dotted lines separate wheat by ploidy. To test the effect of growth at elevated [CO_2] on leaf thickness, a t-test with a Bonferroni-Hochberg end correction (n = 6) was used to compare individual wheat line means, with grey stars indicating significant differences ($P < 0.05$
